# Monthly Summary of Medical Journalism

**Published:** 1860-04

**Authors:** O. C. Gibbs

**Affiliations:** Frewsburg, N. Y.


### ECLECTIC DEPARTMENT,

### AND SPIRIT OF THE MEDICAL PERIODICAL PRESS.

*Monthly Summary of Cotemporary Medical Journalism.* By O. C. GIBBS, M.D., Frewsburg, N. Y.

*Bloodletting in Inflammation, &c.*—In the *American Journal of Medical Sciences*, for January, Prof. L. M. Lawson has an able article upon the *treatment of inflammation*, with special reference *to pneumonia*, in which the views of Dr. Bennett, of Edinburgh, are strongly combated. Dr. Lawson considers the views of Dr. Bennett, in regard to inflammation and its appropriate treatment, inconsistent with clinical experience and well-established pathological principles. We feel a personal interest in this matter, because we stand committed against the use of bloodletting in pneumonia, and our opinions may have been too imperfectly expressed. We have a high appreciation of bloodletting, as a remedy in inflammation, under certain circumstances. We believe, however, that it has been too indiscriminately employed. It is not every inflammation that is best treated by bloodletting, and this is the point for which we have more especially contended. For the last three years, we have not bled in cases of inflammation of any character, and have seen no occasion to regret it. We have not prescribed bleeding because we object to it as a remedy, but because we have not seen the conditions which constitute a demand for it. In that time, we have, perhaps, treated sixty cases of pneumonia, of all ages, without a single fatal issue. We have sometimes given antimony, always Dover's powder, also quinine sooner or later, and generally the veratrum viride. We are of the opinion that, in this locality, we have been, for several years, under a typhoid influence that modifies all diseases. Should we see a high grade of inflammatory fever as was not uncommon ten years ago, we should probably bleed as freely as ever. Many physicians have supposed that all inflammation, especially of the lungs, should be treated with loss of blood, antimony, and perhaps mercury; and that, whatever the age of the patient, or grade of action, quinine was never appropriate in the early stages of the inflammation. It was to combat this error that we have reported cases of pneumonia treated with quinine, opium and ipecacuanha, successfully, with an average duration of less than ten days.

Dr. Lawson considers the statistics of Dr. Bennett altogether unreliable. We have but little confidence in statistical evidence of any plan of treatment.

We do not understand Dr. Lawson as advocating bloodletting in all cases of inflammation, but in opposing the views of Bennett and others, that it is never beneficial, but always prejudicial.

In this view we heartily concur, and the following quotation from Dr. Lawson's paper cannot be too attentively considered: "It is evident, therefore, that a rational treatment must secure to each case its own individuality; and as the shades of differences and the corresponding modifications of treatment cannot be expressed in *groups*, statistics, in this sense, become simply an impossibility. For example, bleeding, antimony, mercury, and blisters may be demanded in one case; quinine, opium, and wine in the next; a third may require but little interference except a well-regulated diet with moderate stimulants; and so on, *ad infinitum*. The treatment of pneumonia demands not a single, but many agents; and he who would attempt to develop results by statistics, will be required to make each group a *unit*. It is the proper *combination* of remedies, and not a single agent or mode of practice, which is capable of securing the best results in the treatment of disease."

While Prof. Lawson admits that there is a lowering of the grade of diseased action of late, which requires less depletion than formerly, yet he is "inclined to believe that the great outcry against bleeding has driven us to the opposite extreme, and we now deplete less than the interests of our patients frequently require." This may be true in some localities; but, as we have observed, physicians have been slow to recognize the lowering grade of diseased action, and have bled beyond the requirements, or, as we believe, the necessities of the present modified forms of disease.

In the *New Orleans Medical and Surgical Journal*, for January, is a paper on *Inflammation*, by Prof. Warren Stone, of New Orleans, that might be profitably read in connection with the paper of Prof. Lawson. We have not space here for an analysis of it, but will take the liberty to quote a pertinent passage or two. Prof. Stone recognizes the propriety of opposite modes of treating inflammation, rendered appropriate, and even necessary, by the circumstances of individual cases. Thus, he says: "In an inflammation, when we can afford to pull down, no great skill is required in the management; but when we are required to build up and lend force to vital actions, much delicacy is required in the management." Further, and more to the point, in regard to the subject of Prof. Lawson's paper, he says: "There is no disease, I believe, in which a fixed routine treatment operates so badly as in pneumonia; and those who have continued the old plan of bleeding, when thought admissible, and giving tart. antimony in the first stage, and calomel in the second stage, have been the most dangerous routinists."

With the above remarks and quotations, we leave two very important contributions to the medical journal literature for January.

*Nitric Acid in Remittent Fever of Adynamic Type.*—In the *American Journal of Medical Sciences*, for January, Dr. Bedford Brown, of North Carolina, has an article upon the adynamic type of remittent fever and its treatment. We quote the substance of his views in regard to treatment. He says: "While a large majority of patients, suffering from attacks of the simple and inflammatory forms of the disease, will recover under the use of such means as local and general bleeding, moderate purgation, sulph. quinia, mercury, oil of turpentine, &c., these remedies have proved utterly inefficient in the adynamic or malignant types." Speaking of nitric acid in such cases, he says: "The tonic properties of the acid are not due simply to any influence which it may exert on the digestive organs. But that it aids in the formation of the blood, and renders it more plastic and organizable, cannot be doubted." * * * "With these considerations in view, I resorted to the use of this remedy in the treatment of remittent fevers of an adynamic type. The powers of the remedy have been satisfactorily tested in at least forty cases by myself and partner, Dr. Roan. The acid was administered in simple cases, in connection with sulphate of quinia and other minor remedies. To the quinia it has certainly added efficacy. A large proportion of the cases were of a decidedly malignant type. In a sufficient number of these to determine its value the acid was administered alone, with the exception of a few remedies of minor importance. I do not remember a single fatal case after its free and constant use. I am also impressed with the belief that it possesses much power to prevent the development of adynamia when given early. Patients, after the use of it, recovered more rapidly and completely than under the usual treatment. Under the influence of the acid it was pleasing to witness the change of form to a more simple character, by a successive clearing up and subsidence of the alarming symptoms."

*Vesico-Vaginal Fistula.*—In the above-mentioned journal for January, W. L. Atlee, M.D., reports a successful case of operation for this troublesome affection, accompanied with remarks in regard to the method of operation. Dr. Atlee's plan is a modification of that of Dr. Sims and of Dr. Bozeman, which modifications he considers improvements. While he ascribes great praise to Dr. Sims' for the introduction of the silver suture, he yet believes the blue iron-wire is the best, possessing "in a high degree every property belonging to silver, while in other respects it is preferable." He uses the button of Dr. Bozeman, with some modifications. The button "has a fenestrum along its centre, proportioned to the size of the fistula." He brings the edges of the wound firmly together before applying the button or plate. With the instrument invented by Dr. Coghill, he secures the coaptation of the raw edges of the fistula, with the greatest certainty, by twisting every alternate suture. Having brought the opposite sides of the fistula into perfect contact, by twisting the alternate sutures, these sutures are brought through the fenestrum, while the remaining ones are carried through holes in the button, tightened and fastened with shot, precisely as in the method of Dr. Bozeman. Another improvement, more of convenience than otherwise, is practiced by Dr. Atlee. Each suture, as it is introduced, is fastened to a transverse thread, which arrangement prevents tangling of the wires, and corresponding ends of sutures are found without exposing the fistula. The figures accompanying Dr. Atlee's article make his plan of operation perfectly comprehensible.

In the *New Orleans Medical and Surgical Journal* for January, Dr. Bozeman has an article upon *urethro-vesico and recto-vaginal fistulas*, in which he holds to different opinions, in regard to metallic sutures, from Dr. Atlee. It may be observed here that Prof. Simpson, of Edinburgh, agrees with Dr. Atlee in his preference for the iron-wire over that of silver. Dr. Bozeman thinks the silver is incomparably superior to the iron-wire for ligature in these operations. He says, "I have never yet seen iron-wire remain in the tissues, even a few days, without being turned black. Not only this: I have always observed, where there was much dragging upon the sutures of this metal, they would cause ulceration, and frequently cut out."

In the *London Lancet* for February, (American reprint,) Dr. Isaac Baker Brown has an article upon the above subject. He has now operated 27 times with but one failure, a success perhaps greater than any other operator. Dr. Brown seems rather indifferent whether the silver or iron wire is used. He thinks, however, he has made great improvement over Bozeman's button, in the use of bar clamps. These clamps he thinks possess many advantages, among which the most important are that the edges of the wound may, with *certainty*, be held "in perfect apposition all along the fistula," and "that, however irregular the opening, you can follow its tortuosity without the slightest difficulty." In the *Nashville Journal of Medicine and Surgery* for February, Prof. Paul F. Eve has an article upon the above subject, in which he proposes a *screw-clamp* and the *twisted suture*. For his method he claims many advantages, but which we have not space at present to particularize.

*Hydrophobia.*—In the January number of the *American Journal of Medical Sciences*, Dr. J. E. H. Ligget, of Middleburg, Md., reports a case of hydrophobia, successfully treated with drachm doses of calomel. The patient was first bled to the amount of 36 ounces, and then drachm doses of calomel were ordered "to be repeated every *four* hours, if the symptoms remain unabated. If the spasms decline in frequency and violence, the intervals to be lengthened to six or eight hours." Ptyalism was well established on the third day. Opium was given occasionally, and quinine to counteract the tendency to exhaustion. The patient was discharged cured on the 11th day of treatment. Dr. Ligget says, "The increased flow of saliva appears to be a conservative effort of the *vis medicatrix* to eliminate the poison from the system, through the glands engaged in its secretion." This opinion forms the basis of his treatment. The theory of its action is of but little importance, if the utility of the treatment be established by subsequent experience.

*Diphtheria.*—In the *New Orleans Medical and Surgical Journal* for January, Dr. S. L. Bigelow has an article upon the above subject. Diphtheria has been a fruitful theme in journal literature for some time past. We have endeavored to keep our readers thoroughly informed in the more important opinions in regard to treatment. Dr. Bigelow thinks "diphtheria rarely occurs as a sporadic disease, almost invariably as an epidemic," and he is strong in the belief that "if there exists a mediately *contagious* disease on earth," it is the disease under consideration. Of its nature he says, "It is evidently a typhoid disease, and as such must be treated from the start; it is a septic disease, and as such must be combated at the earliest moment; it is accompanied by local accidents, and they must be treated as they present themselves."

It would require much space to give Dr. Bigelow's treatment in all its minuteness; as we consider it quite important, we shall, as briefly as possible, give it in substance. He says, "First, then, I advise at my first visit an insufflation of about a drachm of very dry powdered burnt alum, whether I find a commencement of the plastic deposit on the tonsils, or in the pharynx, or not." * * * "At the same visit I also advise a tepid bath of an hour's duration at least," * * * "and a purge of citrate of magnesia." * * * "I prefer the alum as a topic most decidedly to nitrate of silver, as it forms no eschar to blind you at your next visit, with regard to the exact state of things in the throat, and also because I have found it really more potent in its good influence as a local application. The tongue is to be held down with a large spoon, and the alum blown in through a single glass tube, six or eight inches long, and one-fourth of an inch in diameter, at the moment when the presence of the spoon in the fauces causes the involuntary act of gagging on the part of the patient. This insufflation I order to be continued every hour until my return." His first general treatment "consists in the administration, every three hours, of ten grains of the chlorate of potash, and ten grains of the bichlorate dissolved in some convenient vehicle, with the administration ordinarily of one-tenth of a grain of calomel in sugar, dry, upon the tongue, every hour or every two hours, stopping and recommencing its administration according to circumstances, which can only be explained or appreciated by the physician at the bedside." * * "In addition to the above, I commence immediately with the use of tonics, stimulants, and the most nourishing possible fluid animal food. Quinine every three hours, in as large doses as can be borne, without producing severe cerebral perturbation; bitters composed of cinchona, gentian, columbo, chamomile, quassia, bitter orange-peel, &c., formed into a strong infusion, to which I add brandy, and a little syrup." This constitutes Dr. Bigelow's treatment, divested of its minutiæ of detail. We said last month that we should expect benefit from an occasional emetic; Dr. Bigelow prefers to remove the pseudo-membranous deposit with long forceps, or by scraping, &c., instead of by emetics, of which he says, "I have never yet prescribed a single grain of emetic in a case of *angine couenneuse*, and I hope that it may never be given me so to do." Of bleeding he says, "I would as soon put my lancet into the vein of a moribund typhique, as to take an ounce of blood from a patient with membranous sore throat under any circumstances of the disease which I have ever witnessed, or can imagine."

In the *British and Foreign Medico-Chirurgical Review* for January, Dr. J. B. Sanderson has an able article upon the pathology of the disease under consideration. We have not space at command to give it an analysis. The article is illustrated with the microscopic appearance of the pseudo-membranous exudation.

In the *Chicago Medical Journal*, for October, November, January, and February, Dr. W. G. Dyas has an able series of articles upon the subject of diphtheria. Dr. Dyas' views of the pathology of this disease do not differ materially from those of Dr. Barker, of New York, whose opinions were given last month. He says: "In croup, there is increased fibrin in the blood, not a putrid crasis, nor a tendency to it, as in diphtheria: in the one, there is the sthenic type of fever; in the other, a typhoid, or even no fever whatever; the former is not preceded by a depressed, nor followed by a cachectic state; these conditions more or less usher in or supervene on the symptoms of the latter." In the *Journal*, for February, Dr. Dyas enters rather fully into the subject of treatment. He does not favor the administration of calomel, in any case of this disease, except as a cathartic. In regard to emetics, his opinion corresponds with that expressed by us a month ago, but does not accord with that expressed by Dr. Bigelow, as quoted above. He says: "Emetics are occasionally serviceable, especially in young children, at the period of the invasion of the disease, and when the false membrane extends to the trachea. They should never be omitted when, together with fever, there are foul tongue, want of appetite, and nausea early in the complaint." * * * "Independently of their removing morbid secretions, emetics seem to effect some change in the nervous system that controls and directs the physiological transformations of the blood; and when these transformations are abnormal, the deviation from the healthy state may be corrected or modified by some impression resulting from such change." Dr. Dyas thinks that it is upon stimulants and tonics that we have mainly to depend, in the treatment of the disease under consideration. He says: "The best tonic, perhaps, that can be used in this complaint, is the bitter wine of iron, as prepared by Mr. Thompson." The following is a summary of his treatment: "Early in the attack, the administration of an emetic—then a gentle purgative of some mild preparation of mercury and rhubarb, and the application of a 30-grain solution of nitrate of silver to the fauces. Immediately after the bowels shall have been moved, tincture of sesquichloride of iron should be commenced with, alternating the doses with port wine and beef-tea, and at the same time the throat may be enveloped in flannel, steeped in some stimulating embrocation. Some prefer a large poultice of chamomile flowers, whilst others object to all moist applications of this kind. Under no circumstances whatever should leeches or blisters be applied, as they not only tend to a still further depression of the system, but are apt to be followed by cutaneous diphtheria."

*Tobacco in Rattlesnake Bites.*—In the *New Orleans Medical and Surgical Journal*, for January, Dr. Peake, of Mississippi, has an article upon the antidotal properties of tobacco over the poison of the rattlesnake. He says the efficacy of this remedy is well understood by planters in Arkansas. His own experience is thus given: "While engaged as a land surveyor, one of my chain-bearers was bitten on the dorsum of the foot by a rattlesnake of the largest kind. It was in the month of August, when the poison of this snake is supposed to be more deadly than at any other time. In less than five minutes I had him chew and swallow near two ounces of common chewing tobacco. It did not cause him to vomit; indeed, so far was this from being the case, that he was not even nauseated by it. His limb swelled but very little, and he was up and about the next day, well."

Under such circumstances, and where Bibron's antidote is not to be had, it would be proper to give the tobacco a further trial. We should, however, about as soon expect recovery to follow the bite, as the administration of *two ounces* of tobacco.

*Endermic Medication.*—Of late, *hypodermic* medication has attracted considerable attention; but perhaps no more than it deserves. It is, however, quite possible that the merits of *endermic* medication may be neglected in the furor for the new and more difficult hypodermic plan. In the January number of the *New Orleans Medical and Surgical Journal*, its very able and industrious editor, Dr. Bennet Dowler, has a lengthy article *On the Modes of Medication.* We have not space for its analysis, though the article is one of much merit. We will, however, not pass the article without referring to one case therein illustratively alluded to. In a case of *delirium tremens*, in which morphine to the amount of three or four grains daily, and subsequently laudanum in tea-spoonful doses, had been administered for several successive days, Dr. Dowler used laudanum externally, with the effect of speedily putting his patient to sleep. For seven days the patient had not slept, notwithstanding these large doses of morphine and laudanum. Dr. Dowler says: "Next morning a drachm of laudanum was rubbed on the epigastrium, and the same quantity three hours afterwards. Unexpectedly, soon after the last application, he fell into a good sleep. In two days after, he left his bed."

We are confident the endermic method of administering remedies is too much neglected, especially in some cases of gastric irritability.

*Stomatitis Materna.*—In the *Southern Medical and Surgical Journal*, for January, Dr. D. S. Brandon has an article upon the pathology and treatment of the above-mentioned disease. He is of the opinion that the oil of turpentine will be found to be the most efficient of all known remedies. He says: "I have used it, and caused it to be used, in quite a number of cases since the first of last year, when I began its use, and in no instance that I know, or have heard of, has it failed. If the bowels are costive, I premise a dose of caster oil; then give the turpentine, say twelve drops three or four times a day on a little loafsugar. If there be diarrhœa present, I use equal parts of laudanum with the turpentine, as above." * * "The cure is usually effected in from five to eight days; very bad cases may require more time." However decided may be the benefit to the mucous surfaces, resulting from the use of the turpentine in this disease, we should hardly expect to remedy the constitutional impairment, which always accompanies these cases, without the aid of more decided tonics.

*Strychnia in Seminal Emissions.*—In a former number of our *Summary,* we made the following remark: "We are confident that the remedial powers of strychnia are not yet fully brought out." This forms the text of an article, by J. McF. Gaston, M.D., of Columbia, S. C., published in the *Southern Medical and Surgical Journal,* for January. From that article we extract the following: "In that deplorable condition attended with involuntary seminal emissions, I have tested it fully and satisfactorily during a series of years, and it really has served my wishes so completely in these cases that I now use no other course of treatment. With a view to secure the best effects from it, a proper regimen should accompany its use; and with such a course, it may almost be regarded as a specific in spermatorrhœa."

*Wound of the Brain.*—In the *New Orleans Medical News and Hospital Gazette,* for February, Dr. G. Devron reports an interesting case of punctured wound of the brain. The wound was made with a pocket knife, "at a point nearly corresponding to the junction of the sagittal and lambdoidal sutures." The blade was driven completely through the skull and broken off. The patient rested well the first night, and took his meals through the day without complaining. No symptoms of any urgency presented themselves for the first forty-six hours; he then complained of pain in the head, gradually lapsed into a state of coma, and died. On examination, the blade was found "penetrating the brain to the depth of two inches." This case is interesting because of the comfortable condition of the patient for nearly two days after so grave an injury, and because of the sudden death after the first manifestation of pain, or other unpleasant symptoms.

*Tongue Removed by the Ecraseur.*—In the *New Orleans Medical News and Hospital Gazette* for February, Dr. S. Choppin reports a case of removal of the tongue, for cancer, with the écraseur. The operation lasted fifteen minutes, and was accompanied with no hæmorrhage. This operation is usually accompanied with considerable hæmorrhage, and it is highly probable that the écraseur is, in such cases, a valuable surgical appliance.

*Radical Cure of Hernia.*—Dr. Choppin, referred to, is an earnest advocate of the Wurtzer plan of operating for the radical cure of hernia. He has operated many times with success, and has demonstrated, by post-mortem examinations of subjects operated upon years before, that positive occlusion of the canal had taken place; thereby rendering the recurrence of the hernia impossible. The editors of the *Medical News and Hospital Gazette*, referring to Dr. Choppin's operations, and his lecture upon this subject in the Charity Hospital, say, "We have several times before called attention to this most valuable operation, and offer no apology for repeating our opinion, that it is one of the most important surgical innovations of the age, if not absolutely the most important." Several eminent surgeons have ridiculed this operation; but, really, we hope the views and experiences of Prof. Choppin may be proved to be correct by subsequent clinical observation.

*Radical Cure of Hernia.*—In the *Medical Press*, for February 11th, Dr. J. W. Rosebrugh reports a case of hernia apparently cured, after two operations after the plan of Wurtzer. He says: "The inguinal canal was so large that *three* good-sized fingers could be introduced into it." Hopes of success were entertained after the first operation, but after a month the patient felt something give way, and a fold of intestine descended into the scrotum. On reducing the hernia again, "the canal was found to be so small that the point of one finger could scarcely be insinuated into it." Encouraged by a partial success, the operation was repeated, and three months after there is every prospect of a radical cure.

Prof. J. C. Nott, of Alabama, writing from London to the *New Orleans Medical and Surgical Journal*, and speaking of this operation, says: "In Paris, I talked with Velpeau, the Nestor of French surgeons, with Nelaton, and others, and they all say that Wurtzer's operation, or any other on similar principles, cannot be relied on, the disease returning in the great majority of instances. In fact, the operation is scarcely performed at all now in Paris." Opposed to these views, we may instance the following, as the most recent, in addition to those previously referred to. One of the editors of the *New Orleans Medical News and Hospital Gazette*, in the February issue, says: "The fact that the radical cure of hernia can be nearly always accomplished by the method under consideration is no longer to be disputed, and he who sneers at it is only furnishing a stick with which to have his own head broken."

In the *Charleston Medical Journal and Review* for January, Dr. T. L. Ogier reports twelve successful operations by Wurtzer's method, and he says he has performed nineteen other successful operations, not included in his report. Dr. Ogier concludes his report thus: " Recent cases, in subjects under forty years of age, are always successful, and as far as my limited experience goes, quite free from danger."

In the paper of Dr. Rosenbrugh in the *Medical Press*, the author says he was not aware that the operation of Wurtzer had ever been repeated in the same individual. In the *Medical Times and Gazette* for August 6th, 1859, Dr. Redfern Davies reports forty cases of this operation, in *five* of which the operation had to be *repeated.* He says, " Where the rings are very large, and relaxed, the operation is sometimes unsuccessful, and has to be repeated." Out of Dr. Davies' 40 cases, " but two were complete failures, and of these one was owing to supervention of small-pox."

If the operation for the radical cure of hernia is seldom resorted to in Paris, as we are led to believe by reports, it is frequently and successfully performed both in England and America.

*Induction of Premature Labor.*—In the *Louisville Medical Journal* for February, Prof. Henry Miller has an article upon the induction of premature labor and abortion, with cases. We refer to it for the purpose of quoting his method of using the *uterine douche.* He says, " For this purpose an apparatus was constructed according to the directions of the German professor, (Kiwisch,) with only a slight and important variation, consisting of a tin box, ten inches square, holding about four gallons, with an india-rubber tube, twelve feet long, attached to the bottom of the tin box by a screw and nut, and having a metallic tube, six inches long, affixed to its other extremity—the end of the metallic tube being fashioned like the nozzle of the common enema syringe. Instead of arranging the apparatus to act on the principle of the siphon, as recommended by Kiwisch, a stop-cock was adapted to the india-rubber tubing, about two feet from its metallic end. To put the apparatus in operation, the box must be suspended on a nail driven into the wall, near the ceiling of the room, say nine or ten feet above the floor; the india-rubber tubing must be screwed on, and the stop-cock turned, so as to prevent the flow of the water till it is wanted. The patient takes her seat on a stool placed in a bath-tub to receive the water, the metallic nozzle is introduced into the vagina, and in contact with the os uteri, and the tin box having been previously filled with water, the stop-cock is turned, so as to pour a continuous stream upon the os uteri until all the water in the box is discharged." Prof. Miller uses the water warm at first, and, if need be, subsequently alternates cold and warm. In the successful case reported, the *douche* was used but once on the first day. On the second, third, and fourth days, it was used twice each day. On the fifth and sixth days he used warm, and then cold immediately after, using the warm and cold *douche* each twice each day when labor set in.

The fact that the *douche* will sometimes fail in inducing labor, and the number of times it has to be repeated before success crowns the effort, will always operate against this procedure. We prefer the separation of the membranes, which can be done at one sitting, is usually safe when properly performed, and is always successful.

*Influence of the Mother's Mind on the Fœtus in Utero.*—In the *Nashville Journal of Medicine and Surgery* for February, Prof. John M. Watson has an able article upon the influence of the mother's mind on the fœtus in utero. He believes the mother's mind may, under certain circumstances, produce changes in the organization of the embryo in her womb, and the reasons for his faith are well stated. We have not the space to follow his arguments, and will content ourselves with stating his fifth and sixth propositions: "5. The mind, when improperly employed, may develop premature puberty, premature ova, and premature menstruation, to which may succeed premature conception; and all the energies of both soul and body being directed to this uterine function, may not certain images, under strong mental excitement, be transferred to the embryo? Seeing that the mind can exert such remarkable and undeniable influences over the female organism, may I not add a sixth fact? 6. That the embryo is sometimes altered or changed in its organization in various ways, through the mother's mind or imagination; or shall we admit the influence of the mother's mind over her in every other respect, and then exempt the organization of the embryo from its influence?"

*Transfusion.*—In the *Chicago Medical Journal* for February, Prof. Brainard reports a case of amputation of the thigh in a feeble subject, in which life was temporarily saved by transfusion of blood. He says: "In order to obviate the danger from loss of blood, I had an assistant hold a bowl under the member at the moment of the incision; in order to catch the blood, and stir it, so as to separate the fibrin, the bowl was placed in another containing water at the temperature of 98°. When the bone had been sawed, and the small vessels secured, the patient appeared to be dying from syncope. I then fixed the tube of the transfusion syringe into the femoral artery where it had been divided upon the stump, and the syringe at the proper temperature; I charged it with the defibrinated blood, which had been actively agitated, so as to be of a bright red color, and threw gently into the artery nearly all the blood which had been secured. A light tremulous movement followed, and for about a minute no change was perceptible; but at the end of this time the respiration and the action of the heart became more regular, and he was soon as well as before the operation. The blood thrown into the artery was just two ounces." We have space only to quote another practical remark in this connection. Prof. Brainard says: "The choice of the artery instead of a vein for the transfusion was made for these reasons: 1st. It obviates the danger of fibrinous clots passing to the heart. 2d. It diminishes the danger of air-bubbles passing to the heart. These are the great dangers of transfusion."

*Chronic Rheumatism.*—In the *Cleveland Medical Gazette* for February, Dr. G. L. Purdy has an article upon the treatment of chronic rheumatism with the *sulphurous vapor baths.* We give Dr. Purdy's method of using the bath: "I had my patient stripped to the skin, and seated on a tight-bottomed chair, thickly enveloped with flannel blankets from the shoulders downward. Place the blankets tightly around the neck, and have them fit snugly to the floor, around the patient's chair, but as far from it as you conveniently can, so there will be no danger of their taking fire from the flames of the sulphur. Now place under the patient's chair an iron vessel containing one-fourth of an ounce of sublimed sulphur of the shops, and upon the sulphur place a small piece of red-hot iron, and combustion immediately takes place. If the vapor should escape through the blankets too much, and thus annoy the patient, place more covering around him, or keep the vapor from the face with a fan.

"The patient should be thus vaporized 20 or 30 minutes, or until a very free perspiration is got up. If the sulphur burns out too soon, add some more. When the bath is discontinued, have the blankets removed, and the patient well sprinkled with half a gallon of cold salt water, and thoroughly dried with brisk friction, and placed in bed, warmly covered, for three hours. The bath should be used once every second day."

Dr. Purdy reports one case that resisted thorough treatment for five months, at the hands of all kinds of physicians, that was completely cured in two months, under the use of the sulphurous vapor baths, with the addition of no medicines, except what had previously been thoroughly and ineffectually tried. This is not new treatment; it has been recommended previously by several good practitioners; but it is probable that it is too much neglected in this country. As an article of medicine, it is probable that sulphur receives too little attention. The late Prof. Physick used to say, "If sulphur were a dollar a pound, it would be a very popular medicine with the medical profession."

*Water Dressings.*—In the *Louisville Monthly Medical News*, for January, Prof. J. B. Flint has a very interesting article upon *water dressing in surgical practice.* We allude to this paper because it is well worth a thorough study. We have long since been convinced that patients have been unnecessarily troubled with counter-irritants, with the ostensible object of preventing suppuration, perhaps as often with injury as benefit. In synovitis and textural inflammations, we think that the soothing influence of aqueous vapor will prevent suppuration quite as often as blisters, &c. We would not be understood as objecting to blisters, and counter-irritation in general, but to the too exclusive resort to such means to the neglect of the more comfortable applications of appropriate water dressings.

Prof. Flint is much in favor of the "vapor dressings," and we quote the following as a very good way of applying them. He says: "No particular, in the details of the antiphlogistic medication we are considering, is more essential to its successful administration, than the careful expression of all superfluous water from whatever substance is used as the means of evaporization. We cannot too explicitly insist upon the understanding, that it is not the application of *water* that we prescribe, but *the vapor of water.*" * * * "An estimable lady, as ingenious as she is assiduous and gentle, in attentions to her sick friends, exhibited to me, a few months since, an improved method of preparing the epithem, in warm water or vapor applications. The folded flannel, of proper dimensions, is to be wrung out of cool or tepid water, and being spread out on a firm surface, the heated flat-iron of the laundress is moved over it till it becomes a receptacle of hot moisture, the nurse or attendant being thus spared the scalding and hurry attending this portion of duty to the sick, when performed in the usual way." We have often applied the wetted cloths to the clean top of the cook-stove, thus filling the cloths with hot moisture, with less trouble than in any other way. The wet cloths should always be covered with some retentive covering, or, what is better, oiled silk. .' One of the most common mistakes respecting the practical use of water dressings consists in confounding their action with that of poul tices." In applying the *vapor dressing* to wounds, he says: "A fold or two of patent lint, thoroughly moistened with hot water, are pressed or wrung out, and quickly laid upon the diseased surface; a piece of oiled silk is immediately, but lightly, placed over it, and the dressing is complete."

*Scarlet Fever.*—Prof. J. W. Benson, junior editor of the *Louisville Medical News*, in the January number of that journal, has the following in regard to the treatment of scarlet fever: "In twenty-five successive cases of this disease, which have been latterly under my professional care, the treatment consisted in inunction of the parotid and submaxillary regions by an unguent composed of fifteen grains of the extract of belladonna to an ounce of simple ointment. This was applied freely and frequently as soon as the patient complained of sore throat. A piece of flannel was afterwards applied, and in no case was any other treatment adopted, except the administration of small quantities of neutral mixture during the day. In some cases of rapidly occurring tumefaction of the throat, the prompt subsidence thereof under the treatment left no room for doubt as to its efficacy. I do not pretend to offer this mode of treatment either as a cure for scarlet fever or as the sole means to be relied upon in any case, but I do claim for it a controlling power over the engorgement, and hence a prevention of those destructive ulcerations of the throat which are so much and so justly dreaded." From what we know of the effects of belladonna in the treatment of mammary inflammation, we should certainly expect benefit from this treatment in the glandular symptoms of scarlatina. In a severe form of any disease acknowledged to be constitutional, as is scarlet fever, we should never have sufficient confidence in any local treatment to the neglect of constitutional means. As an auxiliary to remedies of well-established utility, we hope much from the suggestion of Prof. Benson.

*Asthma.*—In the *Louisville Medical News*, for February, Dr. W. H. Newman has a paper upon asthma, in which some opinions are expressed differing somewhat from those usually received as true. He says he cannot believe that the dyspnœa, which is called asthma, "can be produced by a spasmodic constriction of the pulmonary air-tubes. I cannot believe that those tubes have the power of contracting by spasm. In fact, I doubt even if those tubes possess any muscular fibre whatever, entering into their composition." He expresses his belief that "asthma is not produced by a spasmodic constriction of the pulmonary air-tubes, nor by any nervous disorder, but is owing to some disordered state of the mucous membrane lining the air-tubes, and probably the pulmonary vesicles also, by which the free entrance and exit of the oxygen and carbonic acid are hindered or prevented." He promises to give his views of the true pathology of this disease in a future paper, in which event we shall take pleasure in bringing the results before our readers.

*Treatment of Epilepsy.*—In the *Southern Medical and Surgical Journal*, for February, Dr. Trent, of Richmond, Va., reports two cases of epilepsy cured with the *hydrocyanate of iron.* His formula is the following:

℞.—Hydrocyanate ferri, ʒj.

Pulv. valerianæ, ʒij.

Make into pills, No. 120. Dose, one pill three times per day, gradually increased to four pills a day."

This treatment is not original with Dr. Trent, but, if we rightly remember, was first used successfully by Dr. McGugin, of Keokuk, Iowa. To the above formula one drachm of extract of cannabis indica is added by Dr. McGugin.

*Dysentery.*—In a clinical lecture in the Pennsylvania Hospital, by Francis G. Smith, as per report in the *Medical and Surgical Reporter*, saline cathartics are recommended. We quote from the *Reporter:* "In his own practice he had preferred to use Rochelle salt. Of this one ounce was to be dissolved in a pint of cold water, and a wineglassful taken every hour until a bilious evacuation was produced. It was then to be stopped, and ten grains of Dover's powder given. This, in most cases, was followed by the cessation of the disease." * * * "Of the fact there was no question, that the saline treatment had proven more successful in his hands than any other."

Dr. Smith says that the treatment of acute dysentery was first proposed by a French physician some years ago. This may be so; but if so, the Frenchman's claim to originality must date back at least a quarter of a century.

In the spring of 1855 it was communicated in a letter to Prof. George Mendenhall, by Dr. D. B. Dorsey, both of Cincinnati. Dr. Dorsey said he had treated dysentery with saline cathartics, as specified in the communication, for more than *twenty years.* Dr. Dorsey learned the peculiar plan of treatment from Dr. F. Lemoyne, of Washington, Pa. Dr. Mendenhall published Dr. Dorsey's letter in the *Western Lancet*, for June, 1855. Ever since we commenced to treat disease, we have treated dysentery with cathartics and opiates. On reading Dr. Dorsey's letter, we were favorably impressed with the propriety of the proposed treatment, and disposed to give it a trial because he had used it for 20 years, and in two or three severe epidemic visitations of that disease, with only one fatal case. We have ever since relied upon it, especially in adults, and previously, on two occasions, have commended it to the profession.

As the formula under consideration differs somewhat from that used by Dr. Smith, and because it may be new to many of our present readers, we subjoin it here:

"℞.—Saturated solution sulph. magnesia, f. ℥vij.
Aromatic sulphuric acid, f. ℥j.

Mix." * * "The medium dose of this medicine for an adult is one table-spoonful, diluted with two or three ounces of water, every four to six hours, until it gently moves the bowels." * * "Accompanying each dose, when the pain and tenesmus are great, one-sixth of a grain of sulph. morphia may be given." The dose of the mixture and of the morphine will both require variation to meet the wants of individual cases.

*Scarlatina.*—In the *Journal of Materia Medica*, for February, Dr. Joseph Bates has an article upon scarlet fever. We quote the following: "Dr. McGugin, of Iowa, in referring to the use of ammonia in this disease, says that instead of it, he has used, in an epidemic of scarlatina, during the present winter, chlorate of potassa, in combination with veratrum viride; it was of the anginose variety, and every case was controlled in a few days. He gave the veratrum with the solution of the chlorate, each in doses appropriate to the age of the patient, using the salt as a gargle. Dr. Bostwick mentions a number of cases attended with high fever, and pulse 120 to 140; some of them delirious on his first attendance. He treated all with veratrum and tonics, also applied sinapisms to the throat. In some, attended with cough and excessive secretion of mucus, he vomited the patient by one or two additional drops, with great relief. In several cases of this disease, which the writer has treated within a few weeks, all successfully, without witnessing one unfavorable sequence, he has relied mainly upon *veratrum*, *quinine*, citrate of iron, brandy or whiskey, nitrate of silver, and chlorate of potassa.

